# Parents caring and sham-feeding their child born with Esophageal atresia at home while waiting for reconstructive surgery

**DOI:** 10.1007/s00383-024-05839-1

**Published:** 2024-09-29

**Authors:** AnnaMaria Tollne, Elin Öst, Tuva Nilsson, Markus Almström, Jan F. Svensson

**Affiliations:** 1 Department of Pediatric Surgery, Karolinska University Hospital, 171 76 Stockholm, Sweden; 2 Department of Women’s and Children’s Health, Karolinska Institutet, Stockholm, Sweden

**Keywords:** Esophageal atresia, Delayed reconstructive surgery, Sham-feed, Homecare, Parents

## Abstract

**Purpose:**

For children with Esophageal atresia who have to wait for reconstructive surgery, long hospital stay, delayed introduction of oral feeds and hampered oro-motor function has traditionally been draw-backs for this treatment as the patients have minimal training of oro-motor function while waiting for surgery. In this paper, we present the concept of sham-feed at home awaiting reconstructive surgery with the aim to obliviate these problems. The aim was to describe the characteristics of patients with Esophageal atresia waiting for reconstructive surgery sham-feeding at home by their parents and further describe adverse events that arose.

**Methods:**

The study is a retrospective descriptive single center study on all children with a delayed reconstruction of Esophageal atresia who was sham-fed by their parents at home before reconstructive surgery between January 2010 and January 2023 at the Karolinska University Hospital, Stockholm.

**Results:**

Nine patients where home waiting for reconstructive surgery for a median of 72 days. No adverse events were reported related to the sham-feed procedure at home. The children had reconstructive surgery at a median 120 days of age. Five of the children ate full meals orally day 8–27 after surgery. Two children ate fully before 1 year after surgery. Two children had surgery less than 1 year ago and were not eating fully orally at the time of data collection.

**Conclusion:**

Sham-feeding at home by the parents was safe and feasible with the benefits of a prolonged time out of hospital awaiting reconstructive surgery.

## Introduction

Esophageal atresia (EA) is a rare congenital malformation with a prevalence of 2.43/10.000 newborn children [1]. The esophagus is not in continuation from the mouth to the stomach in the child, hence the child cannot swallow saliva or milk after birth. In most cases, the child can undergo surgery with restoration of the esophageal continuity in the 1st days of life, but about ten percent of the children need to wait for reconstructive surgery. This may be due to factors such as prematurity, long gap between the segments of the esophagus (LGEA), or associated anomalies such as advanced cardiac malformations. In these cases, it may be beneficial for the child to wait for a delayed reconstruction. Fifty-three percent of children with EA also have associated anomalies and thirty-five percent are born prematurely [2]. When the child need to wait for surgery, the esophageal segments may grow and this, together with the sturdiness of a more mature child, makes it possible to perform a delayed primary anastomosis in many cases. Other options are a gastric transposition or a jejunal interposition. Both these reconstructions are also preferably performed on a larger infant. While waiting for reconstructive surgery the child gets a gastrostomy the first days of life for enteral feeding and a tube in the upper pouch with continuous suction of saliva and mucus [3].

Today more than 90% of the children born with EA survives. Along with the decrease in mortality over the years, there is a shift to focus on the importance of major postoperative complications and long-term morbidity [4]. Morbidity can be striking in children with LGEA with high prevalence of gastroesophageal reflux (GER), anastomotic strictures, esophagitis, respiratory problems, impaired quality of life and dysphagia [5, 6]. It has also been shown that children with LGEA risk mental health problems during growing up [7]. Children with EA are known to have feeding problems after reconstructive surgery and especially children with LGEA [8, 9]. It has been reported that less than 35% of children under 10 years of age with LGEA can eat orally without restriction [10]. The child’s feeding problems also have an impact on the family and many parents can have feelings of severe anxiety when feeding [11–13].

Oromotor function and swallowing is an intricate process where the newborn child has the ability to swallow milk from the very beginning of life. If this process is not stimulated the child may lose the ability to swallow milk and also not develop the oromotor reflexes and functions necessary to protect its airway and to have an effective swallowing function later in life [14]. Children born with LGEA has an increased risk of developing dysphagia compare to children who have reconstructive surgery first day of life [15]. To help children develop their feeding-skills even though the child do not have a continuous esophagus, sham-feed is an option. Sham-feeding is the process of feeding the child orally, aspirate the milk from the tube in the upper esophageal segment with a syringe and feed the milk back through the gastrostomy [16]. By this process the child learns to suck and swallow, to handle a food bolus in the mouth, to protect its airway and also learns the feed-back mechanism of hunger-feeding-satiety, which is beneficial for children who have to wait for reconstructive surgery [17, 18]. Parents of children with EA have described the experience of sham feeding at home as very positive, that sham-feeding reinforce the healthy abilities in their babies [19].

### Stockholm protocol

The newborn child diagnosed with EA at the Karolinska University Hospital is stabilized and assessed as routine. All children receive a short large-bore single lumen esophageal tube (e-tube) in the upper pouch, connected to a mobile, digital continuous suction device, normally on -10 cmH_2_O suction. The e-tube is a single-lumen 6 or 8 Fr tube 40 or 50 cm long. During the first days of life it is decided if the child will undergo immediate reconstructive surgery or a delayed repair. This decision is often made at a gap-assessment and if the decision is delayed repair, the child will receive a feeding gastrostomy. In case of an upper fistula, this needs to be closed prior to the initiation of sham-feed.

**Fig. 1:**
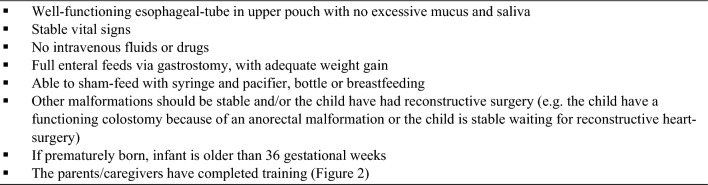
Child status when going home with parents before reconstructive surgery

**Fig. 2:**
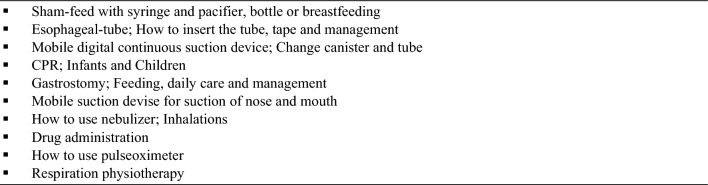
Training for both parents before going home

Already, a few days after the decision to perform a delayed repair the parents/caregivers are introduced to sham-feeding and also to sham-feeding in the home setting. The initiation of sham-feed is depending on the individual child and its signals. The decision to initiate sham-feed is a joint decision between the surgeon and the nurses, but the timing is up to the nursing team. The process starts with breastmilk or formula in the corner of the mouth while the child sucking on a pacifier. The child needs to be alert, showing a strong sucking reflex and the e-tube needs to be working well with no excessive saliva and mucus in the mouth. If the initial sham-feed is tolerated with no signs of respiratory distress, the child can start sham-feeding by bottle or breast, depending on the preference of the mother, and the volumes are increased as tolerated. Initially, the process is supported by two nurses together with one parent. The parent is feeding the infant, one nurse is aspirating the milk through the e-tube with a syringe and the second nurse is feeding the milk back to the child through the gastrostomy in the same pace as the child is swallowing. The volumes are depending on the individual child and may change from day to day. As the teaching process continues, the second parent/caregiver gets involved and after a while the two parents/caregivers perform this process on their own. We recommend the parents to read their babies cues when hungry and sham-feed accordingly, but never sham-feed at night. Before consider the parents to go home with their child, the child should meet some criteria (Fig. 1) and the parents should feel comfortable with all their child’s nursing needs including sham-feeding and completed training (Fig. 2).

During the time sham-feeding at home, the child comes regularly to the pediatric surgery ward at the Karolinska University Hospital or to their pediatrician or nurse at their local hospital, to monitor the child’s nutritional status and to assess how the parents and child manage at home. The parents change the e-tube by themselves at the hospital, as much as possible, to maintain skill. If the child and family live too far away from Stockholm for weekly visits, the EA-nurse from the Karolinska University Hospital will call every week the first months for support and answer questions. Parents can also always call to the pediatric surgery ward at the Karolinska University Hospital for advice.

The aim of this study is to describe the characteristics of children waiting for reconstructive surgery, sham-feeding at home by their parents and further describe adverse events that arose.

## Methods

### Study design and participants

This study is a retrospective descriptive single center study on all children with a delayed reconstruction of EA who was sham-feeding at home before reconstructive surgery between January 2010 and January 2023 at the Karolinska University Hospital, Stockholm. The Karolinska University Hospital is a tertiary referral center, the largest in Sweden and since 2018 one of two centers for EA repair in the country. The annual case load of children born with EA is 9–27 cases. Electronic medical records were reviewed. Children who had their first surgery at another hospital and then referred to Karolinska University Hospital for further care where excluded. So was also a child with gastric transposition due to complications after initial primary repair. The whole cohort of children waiting for reconstructive surgery is briefly presented for reference.

### Baseline characteristics

Gender, gestational age, birthweight and -length, type of EA, associated malformations and other anomalies were collected from the medical records.

### Pre- and postoperative data

Data regarding nutrition and sham-feeding were obtained as well as type of e-tube and management of upper airways: inhalation therapy and if high flow oxygen was being used. Days at Neonatal intensive care unit (NICU), Pediatric intensive care unit (PICU), High dependency unit (HDU), pediatric surgery ward and total hospital length of stay. Data of adverse events as well as reason for readmission were collected as well. Data of postoperative nutrition; date of first enteral- and oral feeding and time of full oral meals were collected.

### Surgical data

The child’s age at the time for reconstructive surgery, type of surgery and preoperative complications were obtained. Data were also collected on surgeries for other malformation.

### Statistical analyses

Descriptive statistics were used to characterize patients, described as numbers (percentage). Nonparametric variables are presented as median and range.

### Ethical approval

Ethical approval was granted by the Swedish Ethical Review Authority, register numbers 2023-00384-01 and 2023-02649-02. The patients were identified through the hospitals quality database. Written information of this study was sent by mail to the parents of all patients who underwent a delayed EA repair at our institution between 2010 and 2023. Signed informed consent were obtained before the patient were included in the study. After data collection, and before the data analysis, the patient data were anonymized.

## Results

**Fig. 3:**
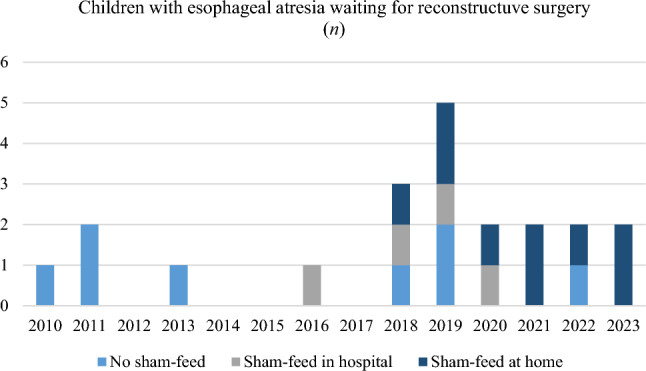
Children waiting for reconstructive surgery distributed over time

Between January 2010 and January 2023, a total of 190 patients were enrolled in the EA treatment program at the Karolinska University Hospital. Twenty-three infants needed to wait for reconstructive surgery due to a long gap, prematurity or other malformations. Twenty-one signed informed consent to participate in the study. A total of thirteen infants sham-fed before surgery and nine sham-fed supported by their parents at home. The development of sham-feeding at hospital and at home over time is presented in Fig. 3. Baseline characteristics is presented in Table 1.

**Table 1:** Baseline characteristics of patients with Esophageal atresia at the Karolinska University Hospital between January 2010—January 2023

	All children born with EA *n* = 190	Children with delayed reconstruction *n* = 21^a^	Children sham-feeding at home awaiting reconstruction *n* = 9
Girls *n* (%)	79 (42)	10 (48)	4 (44)
Birth weight grams, Median (range)	2702 (665–4600)	1896 (665–3364)	1553 (1135–3364)
Low birth weight (< 2500 g) *n* (%)	78 (41)	14 (67)	5 (56)
Gestational week, Median (range)	37 (24–42)	33 (24–41)	33 (28–38)
Prematurely born (< 37 gestational weeks) *n* (%)	78 (41)	16 (76)	5 (56)
Classification of EA			
Gross A *n* (%)	12 (6)	8 (38)	6 (67)
Gross B *n* (%)	3 (2)	3 (14)	1 (11)
Gross C *n* (%)	160 (84)	10 (48)	2 (22)
Gross D *n* (%)	3 (2)	0	0
Gross E *n* (%)	12 (6)	0	0
Associated malformations and other anomalies* n* (%)	146 (77)	19 (90)	8 (89)
Cardio-vascular *n* (%)	57 (30)	8 (38)	2 (22)
Anorectal *n* (%)	21 (11)	4 (19)	2 (22)
Uro-genital *n* (%)	21 (11)	2 (10)	2 (22)
VACTERL^b^ *n* (%)	91 (48)	8 (38)	4 (44)
Other anomalies^c^ *n* (%)	93 (49)	13 (62)	4 (44)
Genetic disorder *n* (%)	22 (11)	1 (5)	1 (11)
Type of surgery			
Primary anastomosis *n* (%)	150 (79)	0	0
Delayed primary anastomosis *n* (%)	13 (7)	13 (62)	5 (56)
Partial gastric pull-up *n* (%)	11^d^ (6)	6 (29)	2 (22)
Gastric transposition *n* (%)	3 (2)	1 (5)	1 (11)
Jejunal interposition *n* (%)	1 (0.5)	1 (5)	1 (11)
Colon interposition *n* (%)	0	0	0
H-fistula *n* (%)	12 (6)	0	0

^a^Two children declined participation in the study

^b^Children diagnosed with VACTERL association have at least three characteristic features’ of following: vertebral defects, anal atresia, cardiac defects, tracheo-esophageal fistula, renal anomalies and limb abnormalities

^c^Other malformations and anomalies: Cardio-vascular (e.g. mitral regurgitation, arrythmia), gastrointestinal (e.g. pyloric stenosis), urogenital (e.g. undescended testis), cleft and lip palate, limb, vertebrae-rib, eye, ear, central nervous system or respiratory anomaly including tracheomalacia

^d^Four children had partial gastric pull-up first days of life. Seven children had delayed operation and then partial gastric pull-up

### Preoperative nutrition and sham-feeding

All children got a gastrostomy during their first days of life and started enteral feeding. In the group of children sham-feeding at home sham-feeding was initiated at gestational week 33–41, 7–90 days after birth (Table 2). Start of sham-feed depended on prematurity, general well-being and/or symptoms of other malformations or anomalies. Five children sham-fed full meals and one child almost full meals (75% of a meal) before reconstructive surgery, although the amount per meal could vary on daily basis depending on other symptoms. Three children had more difficulties with oral feeds and sham-fed 2–30 ml irregularly and not every day. These children had other severe malformations with significant symptoms that dominated the child’s care and well-being.

**Table 2:** Patients sham-feeding at hospital and home

	Children sham-feeding at the hospital before reconstructive surgery *n* = 4	Children sham-feeding at home before reconstructive surgery *n* = 9
Age start sham-feeding (days) Median (range)	13 (8–84)	27 (7–90)
Gestational age start sham-feeding (Gestational week) Median (range)	39 (31–43)	39 (33–41)
Sham-feeding with syringe and pacifier *n* (%)	1 (25)	1 (11)
Sham-feeding by bottle *n* (%)	1 (25)	4 (44)
Sham-feeding by breast and bottle *n* (%)	2 (50)	4 (44)

### Esophageal tube and managing saliva

All children who sham-fed at home had an e-tube in the upper segment, a single-lumen, 6 or 8 Fr, 40–50 cm long, connected to a mobile, digital continuous suction device as described above. Most children (*n* = 7) had their e-tube changed once a week and/or when increased salivation or dislocation. Two children had their e-tube changed only when needed. Eight children had inhalations with saline in a nebulizer when needed and performed respiratory physiotherapy for mobilization of saliva. Two children also had inhalations with Budesonid once per day. No child was in need of oxygen or high-flow airway support at home.

### Preoperative days in intensive care unit/high dependency unit /pediatric surgery ward

Five of the children were born prematurely (gestational week 28–33) and spent 15–88 days in the NICU. The remaining four children born gestational week 36–38 spent 2–30 days in PICU. Eight children spent 10–93 days in the HDU and one child did go straight to the pediatric surgery ward. Nine children spent 2–69 days in the pediatric surgery ward. Table 3 shows this distribution in all children awaiting reconstructive surgery.

**Table 3:** Distribution of days in and out of hospital for patients with delayed repair

	Children not sham-feeding before reconstructive surgery *n* = 8	Children sham-feeding at the hospital before reconstructive surgery *n* = 4	Children sham-feeding at home before reconstructive surgery *n* = 9
	Median (Range)	Median (Range)	Median (Range)
NICU/PICU (days)	67 (14–119)	25 (2–54)	22 (2–88)
HDU (days)	18 (0–69)	53 (0–75)	29 (0–93)
Pediatric surgery ward (days)	0	14 (0–23)	20 (2–69)
Home (days)	0	0	72 (7–246)

### Sham-feeding at home before surgery

Six parents got the standardized training that was implemented in year 2020 (Fig. 1). Time spent at home varied greatly between the children. One child spent 7 days at home and then readmitted to the pediatric surgery ward due to leaking gastrostomy and problems related to the colostomy. Three of the children born prematurely were home 14 to 72 days waiting for reconstructive surgery, while four children with other significant malformations or in need of gastric pull-up, jejunal interposition or with very long-gap were home 117–246 days (Table 3).

### Adverse events and readmission to hospital

There were no reported adverse events at home related to the sham feed procedure. One child with tracheomalacia were admitted to hospital for observation and enteral antibiotic treatment for suspected aspiration pneumonia with no clear correlation with oral feeding per se. One other child was admitted five times to hospital for suspected upper respiratory tract infections. Five children were readmitted to hospital for different reasons e.g. leaking gastrostomy and other procedures and operations due to their other malformations.

### Postoperative nutrition and oral feeding

The children who sham-fed at home started eating orally between day 3 and 17 after reconstructive surgery. Five of the children ate full meals orally day 8–27 after surgery. These children had their reconstructive surgery between three and seven and a half months of age. Two children ate fully after 339 and 341 days, respectively. They had reconstructive surgery when they were 3 and 10 months old, respectively. They both had other malformations and needed further major surgery for these malformations or complications during first year of life. They had more difficulty sham-feeding before surgery; only smaller amount irregularly and not every day. Two children had surgery less than 1 year ago and are not eating fully orally at the time of data collection. They had their reconstructive surgery at eight and fifteen months of age, respectively (Table 4).

**Table 4:** All children waiting for reconstructive surgery January 2010–January 2023 enteral and oral intake after reconstructive surgery

	Children not sham-feeding before reconstructive surgery *n* = 8	Children sham-feeding at the hospital before reconstructive surgery *n* = 4	Children sham-feeding at home before reconstructive surgery *n* = 9
Age at reconstructive surgery Days. Median (range)	82 (34–119)	86 (56–109)	120 (89–464)
Start eating orally after surgery Days. Median (range)	19 (7–29)^b^	10 (7–63)	8 (3–17)
Full meals orally after surgery Days. Median (range)	160 (14–488)^a,b^	595 (127–1063)^a^	27 (8–341)^c^
Full enteral feeding after surgery Days. Median (range)	14 (8–27)^b^	14 (4–39)	10 (6–42)
Children eating whole meal orally 1 months after surgery *n* (%)	1/8 (13)^b^	0	5/9 (56)
Children eating whole meal orally 6 months after surgery *n* (%)	3/7 (43)^b^	1 (25)	5/9 (56)
Children eating whole meal orally 12 months after surgery *n* (%)	4/7 (57)^b^	1 (25)	7/7 (100)^c^
Children eating whole meal orally 24 months after surgery *n* (%)	5/7 (71)^b^	1 (25)	7/7 (100)^c^
Children eating whole meal orally 3 years after surgery *n* (%)	5/7 (71)^b^	2 (50)	7/7 (100)^c^

^a^Two children are not eating full oral meals 5 years after surgery

^b^One child died 3 months old

^c^Two children had reconstructive surgery less than one year ago, not eating full meals orally at the time of data collection

## Discussion

In this paper, we have presented our method of sham-feeding children with EA in a home setting, while waiting for reconstructive surgery. Nine patients where home waiting for reconstructive surgery for a median of 72 days. No adverse events were reported related to the sham-feed procedure at home. Five of the children ate full meals orally day 8–27 after surgery. Two children ate fully at 11 month of age. Two children had surgery less than one year ago and are not eating fully orally at the time of data collection.

EA is often a complex disease and there is no gold standard for the treatment of the more advanced cases. Delayed primary repair is one way of dealing with the malformation. The Stockholm protocol has the benefit of avoiding major surgery and anesthetics in the immediate newborn period. One draw-back of this method is that the child does not get a continuous esophagus that would allow the maturation of the swallowing reflex and oromotor function that may hamper further abilities. Our primary aim is a long-term solution that gives the growing child and later the adult the best prospect for a life with as little feeding problems as possible.

Our study show promising results that sham-feeding regularly at the children’s hunger cues can help early oral feeding with full meals after reconstructive surgery as more than half of our patients in the study were fully orally fed within 1 month after surgery. In our setting, this is an improvement. Reasons for this may be several. This is possibly based on an interaction between parent and child where the parent practiced reading the child’s signals, both hunger and satiety, when sham-feeding before surgery. The child, in turn, learns to connect the feeling of being hungry, eating and then being full, which may help oral feeding. Feeding problems and dysphagia for children with EA can have many causes other than lack of practice oral feeding [20, 21]. Children with EA who have to wait for reconstructive surgery can have other malformations, such as heart malformations and respiratory problems which also can cause feeding problems; this may also explain our results where some of the children had trouble sham-feeding and start eating orally after surgery [22, 23].

A feared adverse event before we implemented the protocol was that the children would aspirate milk into their lungs when they sham-fed at home by their parents, but the children in our setting had no adverse event when sham-feeding at home. We hypothesize that this is due to early oral stimulation leading to a good oro-motor function, preventing aspiration even if this cannot be proven in this limited series of patients. One child in our setting with tracheomalacia had suspected aspiration pneumonia and was readmitted to hospital with enteral antibiotics. Even though there were no adverse event reported by the parents to this patient when sham-feeding at home and pneumonia is more common in children with EA and tracheomalacia, there is no way knowing that sham-feeding was a risk factor or not [24]. One child was readmitted to hospital for upper respiratory infection five times, but that is a normal frequency of infections in a year for otherwise healthy infants [25].

Children who have to wait for reconstructive surgery have to spend a long time in hospital which have an impact on quality of life for the families [26]. In our setting the families can go home with their children and live a more “normal” life. Being at the hospital can be stressful for families [27]. Studies of home-care for children with other illnesses has shown that parents prefer being at home and had increased quality of life for the whole family [28, 29]. A previous study in Canada has shown that children with LGEA can be safely cared for at home with help from home care [30]. In our center, we train parents to care for their children and sham-feed them at home by themselves. A collaboration between parents and nursing staff in helping the family have a positive relationship with their baby and gain confidence and skill in their ability to care for their infant before they go home, is important [31]. In a recent study in our centre in Stockholm, we interviewed parents who sham-fed their babies at home about their experiences. They were all positive about sham-feeding their child at home and felt that sham-feeding reinforced the healthy abilities and that it influenced the ability to eat. It also gave a feeling of normal life [19]. The promising results of this study together with the parent´s positive experiences of taking care and sham-feed their baby at home before surgery suggest that our protocol is an attractive treatment alternative for these children.

### Patient perspective

EA can be a devastating malformation and every effort has to be made to minimize both short-term and long-term complications. Traditionally a lot of research has focused on short-term data, not focusing on long-term outcome. Supported by patient representatives and organizations, we believe that we need to focus more on long-term outcome and also the experience of the patient and family.

## Conclusions

Sham-feed at home, by parents alone, was feasible and safe for children awaiting a delayed repair of EA. This method needs to be thoroughly taught to the parents and close support is mandatory to provide the level of safety that is required.

## Limitations

It is a small group of children in this study and therefore not possible to compare or generalize data to other children or settings. Although our results are promising, further studies are warranted to elucidate whether sham-feed in hospital or at home is safe and superior to other regimens for treatment to prevent feeding problems for these children. Further research is needed on long-term morbidity, family impact and parents´ quality of life. This method, The Stockholm protocol, should only be used in centers with significant experience and available resources. The Stockholm protocol is not feasible in every EA-center or countries because there may be restrictions regarding medical insurance and parental leave.

## Data Availability

The data that support the findings of this study are not openly available due to reasons of sensitivity and are available from the corresponding author upon reasonable request. Data are located in controlled access data storage at Karolinska Institutet.
